# Changes in Muscle Activation During and After a Shoulder-Fatiguing Task: A Comparison of Elite Female Swimmers and Water Polo Players

**DOI:** 10.3389/fspor.2022.881582

**Published:** 2022-07-13

**Authors:** Savannah King, Lily Dong, Michelle Caron, Julie N. Côté

**Affiliations:** ^1^Biomechanics of Occupation and Sports (BOS) Lab, Department of Kinesiology and Physical Education, McGill University, Montréal, QC, Canada; ^2^Département des sciences de l'activité physique, Université du Québec à Montréal (UQAM), Montréal, QC, Canada

**Keywords:** electromyography—EMG, shoulder, female athletes, water polo players, swimmers, neuromuscular activation, neuromuscular fatigue

## Abstract

This study compared female athletes with different aquatic sports expertise in their neuromuscular activation before, during, and after a shoulder internal rotation fatigue protocol. Eleven water polo players, 12 swimmers, and 14 controls completed concentric maximal voluntary external and internal shoulder rotations before and after a fatigue protocol consisting of concentric internal rotations at 50% of maximal voluntary contraction for at least 3 min or until reporting a rating of perceived effort RPE of 8/10 or higher. Muscle activation was measured for the maximal voluntary contractions, as well as for the first (T1), middle (T2), and third (T3) minute of the fatigue protocol using surface electromyography (EMG) on pectoralis major, anterior and posterior deltoid, upper and middle trapezius, and latissimus dorsi. Intramuscular EMG was used for supraspinatus, infraspinatus, and subscapularis. Pre-fatigue internal rotation torque was significantly correlated with shorter task duration (*r* = −0.39, *p* = 0.02), with water polo players producing significantly greater torque than controls but having significantly lower endurance. Swimmers demonstrated decreased latissimus dorsi activation at T3 compared to T2 (*p* = 0.020, *g* = 0.44) and T1 (*p* = 0.029, *g* = 0.74), differing from water polo players and controls who exhibited increased agonist activation and decreased activation of stabilizers. Comparing the pre-fatigue to the post-fatigue maximal shoulder rotations, water polo players had decreased activation in subscapularis (*p* = 0.018, *g* = 0.67); all groups had decreased activation in latissimus dorsi (*p* < 0.001), though swimmers demonstrated a large effect (*g* = 0.97); and controls had decreased activation in supraspinatus (*p* = 0.005, *g* = 0.71). Together, these results suggest that sports expertise may be associated with different muscle activation both while and after fatigue is induced. Further research should continue to explore sports-specific patterns of muscle recruitment and fatigue adaptations, as well as if certain strategies are adaptive or maladaptive. This may have important consequences for injury prevention among athletes who perform repetitive overhead movements in their sports and who are susceptible to overuse injuries.

## Introduction

Studies suggest that 35–45% of athletes in overhead sports interrupt training due to shoulder problems, with as many as 75% of swimmers having experienced shoulder pain throughout their athletic career (McMaster and Troup, 1993; Joshi et al., 2011; Aliprandi et al., 2013; Matzkin et al., 2016). This high prevalence calls for more research to increase understanding of the mechanisms leading to injury and, in turn, guide improved preventative strategies. The freestyle stroke is frequently used by both competitive swimmers and water polo players during training. The average swimmer executes ≈30,000 shoulder revolutions per week for 50 weeks of the year (Bak and Faunø, 1997; Weldon and Richardson, 2001; Matzkin et al., 2016). Though water polo players swim fewer kilometers, they also perform large numbers of overhead throws during each training session (Wheeler et al., 2013), which also lead to fatigue. Previous research has identified the scapular stabilizers (supraspinatus, infraspinatus, subscapularis, and middle trapezius) and internal rotators (latissimus dorsi and pectoralis major) as important contributors to the freestyle stroke (Nuber et al., 1986; Pink et al., 1991; Weldon and Richardson, 2001; Bedi, 2011). For water polo, the posterior deltoid, supraspinatus, and middle trapezius muscles play active roles in the cocking phase of the throwing task, and the pectoralis major and anterior deltoid in the follow through (Fleisig et al., 2009; Weber et al., 2014; Yaghoubi et al., 2014). When throwing while in water, the lack of a base of support requires the shoulder joint to produce more force than overhead throwing performed on land (Feltner and Taylor, 1997). Water polo players also swim freestyle in a head-up position, requiring more activation from the upper trapezius than in a competitive freestyle stroke.

Fatigue is considered a major contributor to the rate of injury, as it affects shoulder girdle stability (Dale et al., 2007; Joshi et al., 2011; Matthews et al., 2017). Madsen et al. (2011) found that there was a progressively higher rate of abnormal scapular motion (scapular dyskinesis) in a group of healthy swimmers as their training session went on and fatigue developed. Since the occurrence of scapular dyskinesis is linked to shoulder injuries in swimmers (Bak and Faunø, 1997; Kibler and McMullen, 2003), this indicates that fatigue plays a key role in the development of those injuries. Fatigue can be operationalized as decreased maximal force production capacity, or as changes in muscle activation and/or increased rate of perceived effort while maintaining task performance (Vøllestad, 1997; González-Izal et al., 2012; Enoka and Duchateau, 2016). Force and torque outputs provide methods to measure the functional effects of fatigue on performance and can be quantified using an isokinetic dynamometer (Baltzopoulos and Brodie, 1989; Cools et al., 2003). Measures using electromyography (EMG), have shown EMG amplitude (e.g., root-mean square [RMS]) to increase with fatigue under some conditions (Krogh-Lund and Jørgensen, 1991; Smith et al., 2016). This has been interpreted as increases in the recruitment of more and bigger motor units, as well as in increased motor unit discharge rates to continue performing a task. However, this is typically seen in repeated and/or prolonged submaximal efforts only, whereas in short, high-intensity tasks, fatigue is rather linked with decreases in activity amplitude (Vøllestad, 1997; González-Izal et al., 2012).

Research in the workplace setting has demonstrated that EMG can be used to compare two different groups to identify muscle activation patterns that are associated with increased upper limb injury risk (Goubault et al., 2020). Similar comparisons of muscle activation among athletes have not been made, but kinematic studies of landing and cutting among female soccer and basketball players revealed that athletes had sports-specific movement control patterns that, consequently, could indicate sports-specific susceptibility to injury (Cowley et al., 2006; Munro et al., 2012). The repetitive nature of upper limb actions performed by water polo players and swimmers makes it relevant to also consider the role of fatigue. Indeed, internal rotation torque has been shown to decrease after fatiguing throwing tasks (Ellenbecker and Roetert, 1999; Mullaney et al., 2005). As well, throughout swimming-specific tasks, increases in EMG amplitude for the latissimus dorsi and triceps brachii were observed (Stirn et al., 2011). However, there is yet to be research that examines if shoulder muscle activation or subsequent changes in activation with fatigue differ based on sports expertise. Therefore, the objective of this study was to investigate whether female swimming and water polo training experience is associated with patterns of shoulder muscle activation during and following the inducement of fatigue. Performance measures, such as torque production capacity and endurance, were also compared. We hypothesized that water polo and swimmers would have greater endurance during a fatigue protocol compared to a control group, that torque production would decrease when fatigued, but that there would be differences in muscle activation between the groups.

## Methods

### Participants

Thirty-seven healthy female volunteers were recruited into one of three groups: a control group (CON), an elite water polo group (WP), and an elite swimming group (SW) (Table 1). The control group consisted of individuals with no past training in repetitive overhead sports or activities. The elite athlete groups were recruited through the Institut national du sports du Québec network and local national level aquatics teams. The inclusion criteria for the “elite” groups required athletes to have six or more years of experience competing at the national or international level and be participating in 15 h per week or more of sports-specific training (Swann et al., 2015). Based on the classification criteria outlined by McKay et al. (2022), six WP participants were classified as elite/international level (tier 4) athletes and five as highly trained/national level (tier 3) athletes. For the SW participants, two were classified as elite/international level (tier 4) athletes, seven as highly trained/national level (tier 3) athletes, and three as trained/developmental (tier 2) athletes.

**Table 1:** Participant characteristics.

**Group**	***n***	**Age (years)**	**Height (cm)**	**Weight (kg)**
CON	14	24.7 ± 2.4	166.7 ± 6.8	63.4 ± 9.8
WP	11	22.2 ± 5.6	171.5 ± 6.9	80.0 ± 14.8
SW	12	20.4 ± 2.5	169.1 ± 4.7	68.0 ± 6.3

All participants reported having no injuries involving their shoulder joint within the previous 6 months. The research protocol was approved by the Centre de Recherche Interdisciplinaire en Réadaptation (CRIR-1247-0517) and all participants provided written informed consent before the experimental procedure.

### Instrumentation

Wireless surface EMG (sEMG; Delsys Trigno Wireless EMG, Natick, MA, USA) sensors (Hermens et al., 1999) were placed on pectoralis major, anterior deltoid, posterior deltoid, upper trapezius, middle trapezius, and latissimus dorsi according to SENIAM guidelines and those established by Barbero et al. (2012). Intramuscular EMG (iEMG) sensors were used to record activity in three muscles using paired hook fine-wire electrodes (Natus Neurology, Middleton, WI, USA): supraspinatus, infraspinatus, and subscapularis. The insertion points were consistent with those in a study by Gaudet et al. (2018a). The exact locations of the electrode placements and insertion points can be found in Table 2. All electrodes were attached to the skin using double-sided tape, with additional tape over the sensor, with additional tape over the electrode to reduce any movement during the protocol.

**Table 2:** EMG electrode placements.

**Muscle**	**Electrode Position**
**Surface electrodes**	
Pectoralis Major	Three finger widths medial to the coracoid process of the shoulder.
Anterior Deltoid	Three finger widths distal to coracoid process of the shoulder, in line with the humerus.
Posterior Deltoid	Two finger widths distal to the angle of the acromion, on the line between the acromion and the pinky finger.
Upper Trapezius	Midpoint between the C7 spinous process and the anterior acromion process.
Middle Trapezius	50% between the medial border of the scapula and the spine, at the level of T3.
Latissimus Dorsi	Three finger widths distal and slightly lateral to inferior angle of the scapula.
**Intramuscular electrodes**	
Subscapularis	Three finger widths superior to the inferior angle of the scapula on the medial side. Insertion occurred with the scapula winged to be able to insert the electrode under the scapula.
Infraspinatus	2.5 cm inferior to the midpoint of the spine of the scapula.
Supraspinatus	1.5 cm superior to the midpoint of the spine of the scapula.

**Figure 1:**
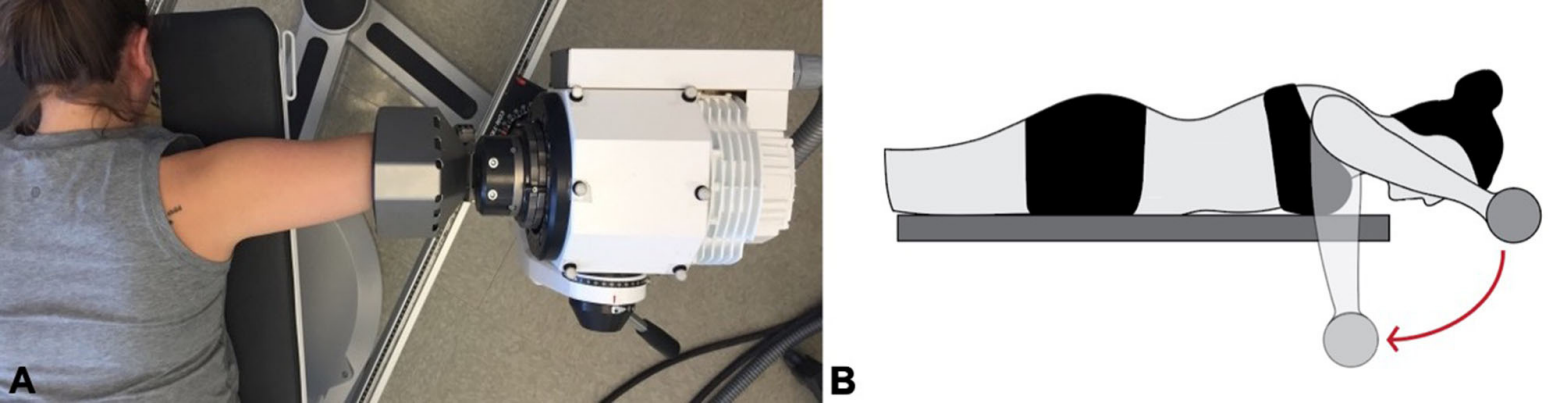
**(A)** Participant setup on the isokinetic dynamometer. **(B)** Movement pattern of the shoulder internal rotation fatigue protocol.

All shoulder rotations were performed using a CON-TREX Multi-Joint Isokinetic Dynamometer (CON-TREX MJ; CMV AG, Dubendorf, Switzerland), with the participant in a prone position on the CON-TREX. The angular velocity of the CON-TREX motor was set to 120°/s to simulate the speed and positioning of a freestyle swimming stroke at peak velocity (Falkel et al., 1987). Figures 1A,B show the positioning and the movement for these trials, respectively. The range of motion varied slightly depending on the participant's flexibility (77.8 ± 5.4°) but remained constant during all of the participant's movements throughout the protocol.

### Experimental Procedure

After the participant's height and mass were measured, the CON-TREX seat and motor were adjusted such that the head of the motor would form a straight line with the participant's glenohumeral joint and elbow. The skin was shaved and cleaned with alcohol before EMG electrodes were placed. Using the dominant arm, the participant then performed a series of concentric maximal voluntary contractions (MVC) with 1 min of rest between each. Four sets of three repetitions were performed: two sets of maximal shoulder external rotations with completely passive internal rotation, and two sets of maximal shoulder internal rotations with passive external rotation.

The fatigue protocol consisted of repetitive shoulder internal rotations at ≈50% of the individual's maximal internal rotation torque. Participants were provided visual feedback throughout: a screen in front of the participant showed a bandwidth of 42.5–57.5% of their maximal internal rotation torque. The instructions were to perform repeated concentric internal rotations, keeping the torque output within the 15% bandwidth, and to allow the CON-TREX to move the arm back to the starting position during the external rotation. The participant performed two rounds of familiarization prior to starting the fatigue protocol. For the first 20 s of each minute, the participant was given verbal feedback by the researchers to remain within the bandwidth. At the 20-s mark, the researchers told the participant to remain constant within the bandwidth for the following 30 s without verbal feedback. In the last 10 s the participant was prompted to rate their perceived effort using the modified Borg CR10 Scale (Borg, 1998). The researcher specified that the perception of effort should be in relation to only the neck/shoulder. The protocol continued for a minimum of 3 min until the participant reported an RPE of 8/10 or higher, or until the torque output fell significantly outside of the bandwidth three times consecutively. This termination criterion was unknown to the participant. Immediately after completing the fatigue protocol, the participant repeated the four sets of maximal voluntary contractions, this time without rest between sets to limit recovery.

### Data Processing

Pre-fatigue and post-fatigue maximal internal rotation and external rotation torque values were identified for each participant. All EMG data were filtered using a zero-lag 2nd order Butterworth bandpass filter (sEMG was bandpassed between 10 and 450 Hz, and iEMG between 10 and 1,000 Hz) and full wave rectified before heartbeats were removed. Additionally, iEMG data underwent a notch filter to remove frequency harmonics. Using a moving window of 100 ms, a peak RMS value was identified for each muscle using the single pre-fatigue MVC contraction that elicited the highest RMS amplitude for the given muscle.

EMG data during the fatigue protocol was partitioned into internal and external rotation using the position data from the CON-TREX. For each internal rotation of the fatiguing task, RMS values were calculated using a 100 ms moving window, and for each muscle, were normalized to the previously determined pre-fatigue maximal rotation peak RMS value. The RMS values for the last five internal rotations before the 50-s mark of the first (T1), middle (T2), and last (T3) minute of the task were averaged to obtain one RMS value for each of the nine muscles. If the task was performed for an even number of minutes, the average of the two middle minutes was taken for T2.

### Statistical Analyses

Because assumptions of normality and homogeneity of variance were violated, a Brown–Forsythe test was conducted to compare task duration between groups, followed by Bonferroni *post-hoc* testing. Separate Generalized Estimating Equations (GEE) were performed on external rotation torque, internal rotation torque, the RMS recorded during the pre- and post-fatigue MVC for each muscle, and the RMS recorded during the fatigue protocol. The GEE models for torque included one within-subject variable (Condition, two levels: pre- and post-fatigue), one between-subject variable (Group, three levels: controls, water polo players, swimmers), and included weight as a covariate. For RMS during the fatigue protocol, the GEE model had two within-subject variables (Time, three levels: T1, T2, T2; Muscle, nine levels) and one between-subject variable (Group, three levels). The GEE model for MVC RMS included two within-subject variables (Condition, two levels; Muscle, nine levels: one for each level) and one between-subject variable (Group, three levels: CON, WP, SW). Pairwise comparisons, with Bonferroni correction, of estimated marginal means were carried out when there were statistically significant main effects and interactions. The magnitudes of differences were evaluated with Hedges' *g* effect sizes.

All analyses were conducted with SPSS (IBM SPSS Statistics for Windows, Version 27.0).

## Results

**Figure 2:**
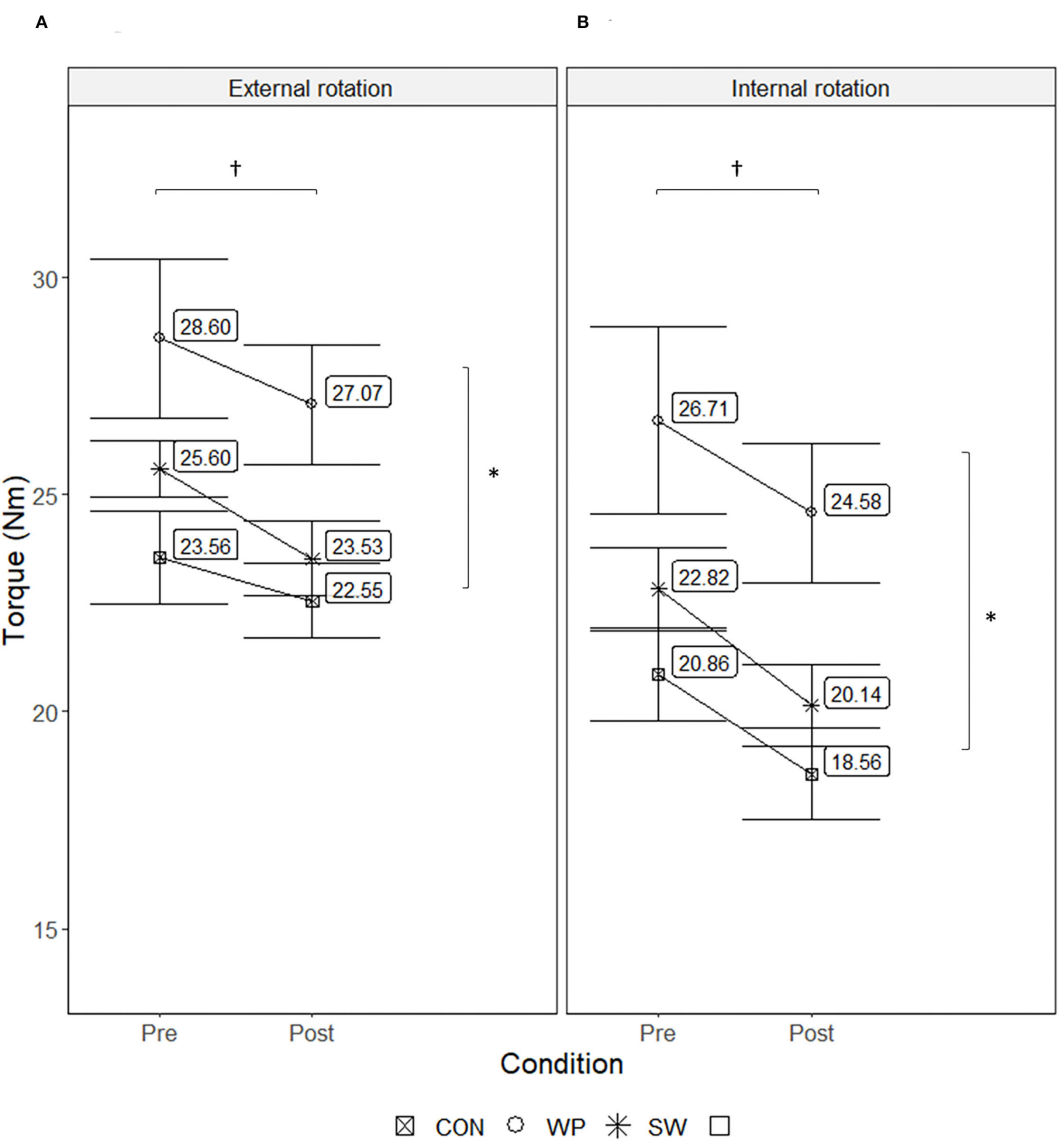
Maximal concentric external **(A)** and internal **(B)** shoulder rotation torque produced by water polo athletes (WP), swimmers (SW), and a control group (CON) before and after a concentric shoulder internal rotation fatigue protocol. Values displayed are estimated marginal means ± SE. †Indicates a significant main effect of the condition, with decreased torque values after the fatigue protocol (*p* < 0.05). *Indicates significant group difference, with WP producing significantly greater torque than CON (*p* < 0.05).

For maximal external rotation torque, there was a main effect of Condition $\left(X_{1}^{2}=10.216,\;p=0.001\right)$ and of Group $\left(X_{2}^{2}=6.826,\;p=0.0033\right)$ (Figure 2A). All groups demonstrated decreased torque in the fatigued condition (*p* = 0.001, *g* = 0.26) and WP produced higher torque than both CON (*p* = 0.031, *g* = 1.64). Similar main effects for Condition $\left(X_{1}^{2}=13.269,\;p<0.001\right)$ and Group $\left(X_{2}^{2}=7.414,\;p=0.0025\right)$ existed for maximal internal rotation torque (Figure 2B), with decreased values after the fatigue protocol (*p* < 0.001, *g* = 0.33) and WP producing greater torque than CON (*p* = 0.020, *g* = 1.81).

**Figure 3:**
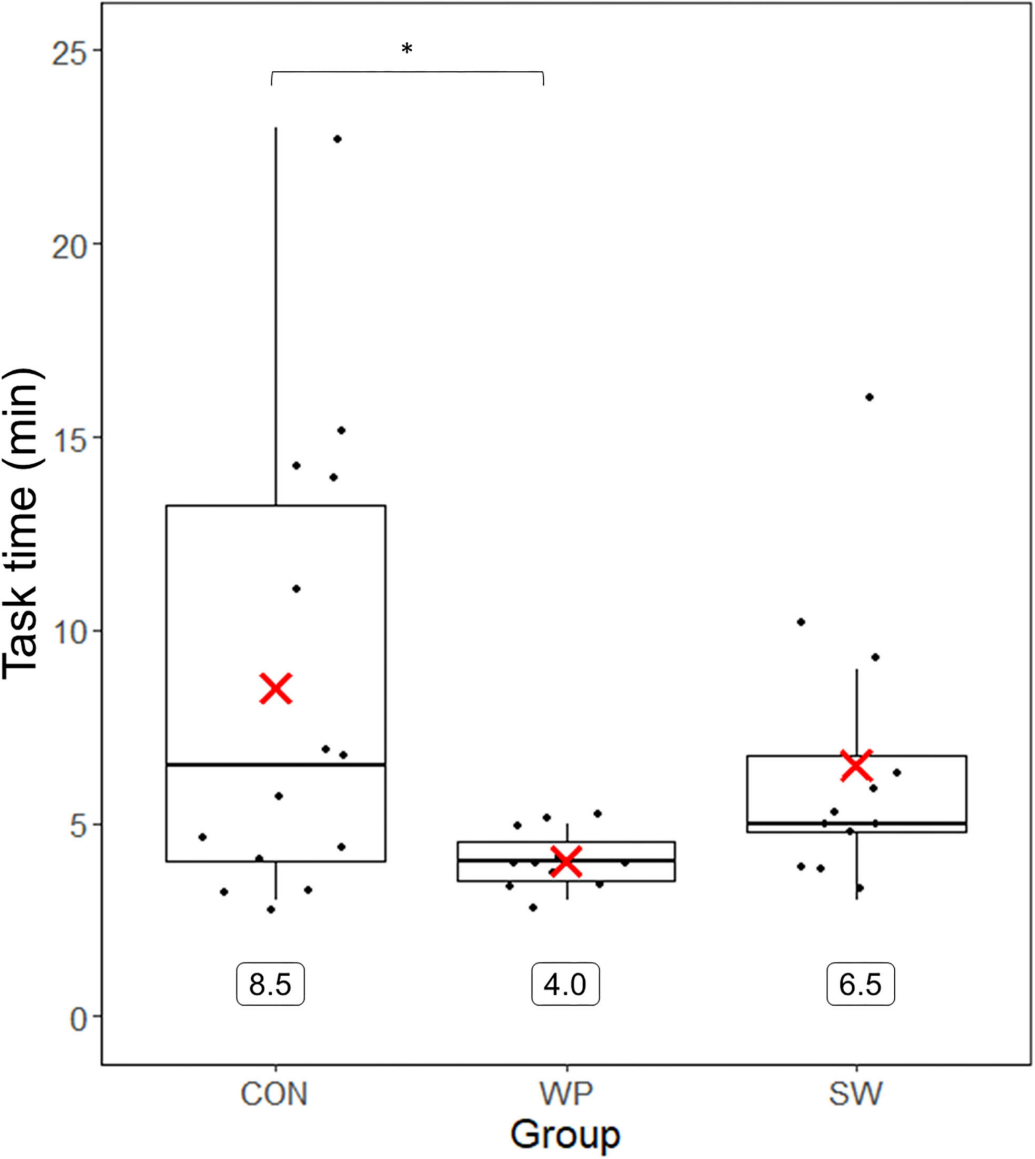
Duration of the fatigue protocol for water polo athletes (WP), swimmers (SW), and a control group (CON). Group means are represented by the red X and displayed numerically below the boxplot. *Significant group difference between CON and WP.

The task duration for CON, WP, and SW was 8.5 ± 6.0 (mean ± SD), 4.0 ± 0.8, and 6.5 ± 3.6 min, respectively. The Brown–Forsythe test indicated differences between groups (*F*(2, 21.828) = 3.917, *p* = 0.035, Figure 3) with *post-hoc* comparisons indicating that CON performed the task significantly longer than WP before reaching the termination criterion (*p* = 0.040, *g* = 0.95). Furthermore, a significant correlation (*r*_s_(35) = −0.39, *p* = 0.02) between maximal pre-fatigue internal rotation torque and task duration indicated that higher internal rotation strength was associated with a shorter time to reach the termination criterion.

**Figure 4:**
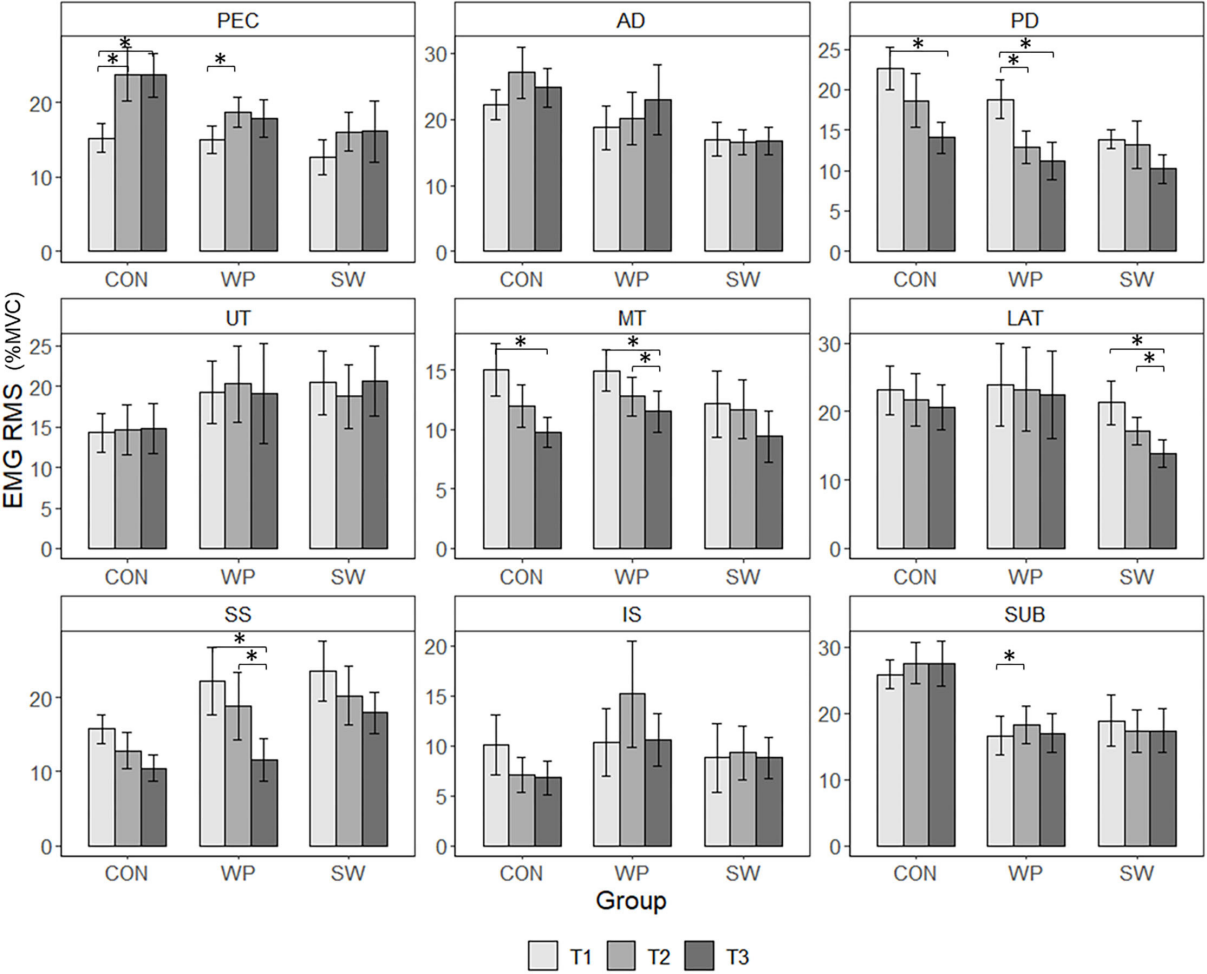
EMG RMS of water polo athletes (WP), swimmers (SW), and a control group (CON) measured during the last five rotations before the 50-s mark of the first (T1), middle (T2), and last (T3) minute of a concentric shoulder internal rotation fatigue protocol. Values are estimated marginal means ± SE measured for pectoralis major (PEC), anterior deltoid (AD), posterior deltoid (PD), upper trapezius (UT), middle trapezius (MT), latissimus dorsi (LAT), supraspinatus (SS), infraspinatus (IS), and subscapularis (SUB). *Indicates a significant group-specific changes in RMS (*p* < 0.05).

During the shoulder internal rotation fatigue protocol, there were significant Group × Time × Muscle interactions for EMG RMS $\left(X_{32}^{2}=315.904,\;p<0.001\right)$ (Figure 4). Among CON, pectoralis major RMS was lower at T2 (*p* = 0.22, *g* = 0.72) and T3 (*p* = 0.015, *g* = 0.85) compared to T1. Also compared to T1, CON had higher T3 RMS for posterior deltoid (*p* = 0.013, *g* = 0.91) and middle trapezius (*p* = 0.023, *g* = 0.72). Among WP, there were increases in RMS from T1 to T2 for pectoralis major (*p* = 0.009, *g* = 0.60) and subscapularis (*p* = 0.012, *g* = 0.16). WP demonstrated decreased posterior deltoid RMS at T2 (*p* = 0.005, *g* = 0.74) and T3 (*p* < 0.001, *g* = 0.90) relative to T1, decreased middle trapezius RMS at T3 compared to T1 (*p* = 0.024, *g* = 0.55) and T2 (*p* = 0.004, *g* = 0.20), as well as decreased supraspinatus RMS at T3 compared to T1 (*p* < 0.001, *g* = 0.85) and T2 (*p* = 0.003, *g* = 0.55). Among SW, only latissimus dorsi RMS changed significantly, with lower RMS at T3 than at T2 (*p* = 0.020, *g* = 0.44) and T1 (*p* = 0.029, *g* = 0.74). At T1, CON had greater RMS for posterior deltoid than SW (*p* = 0.008, *g* = 1.04) and higher subscapularis RMS than WP (*p* = 0.030, *g* = 0.98). At T2, CON had greater anterior deltoid RMS than WP (*p* = 0.048, *g* = 0.45).

**Figure 5:**
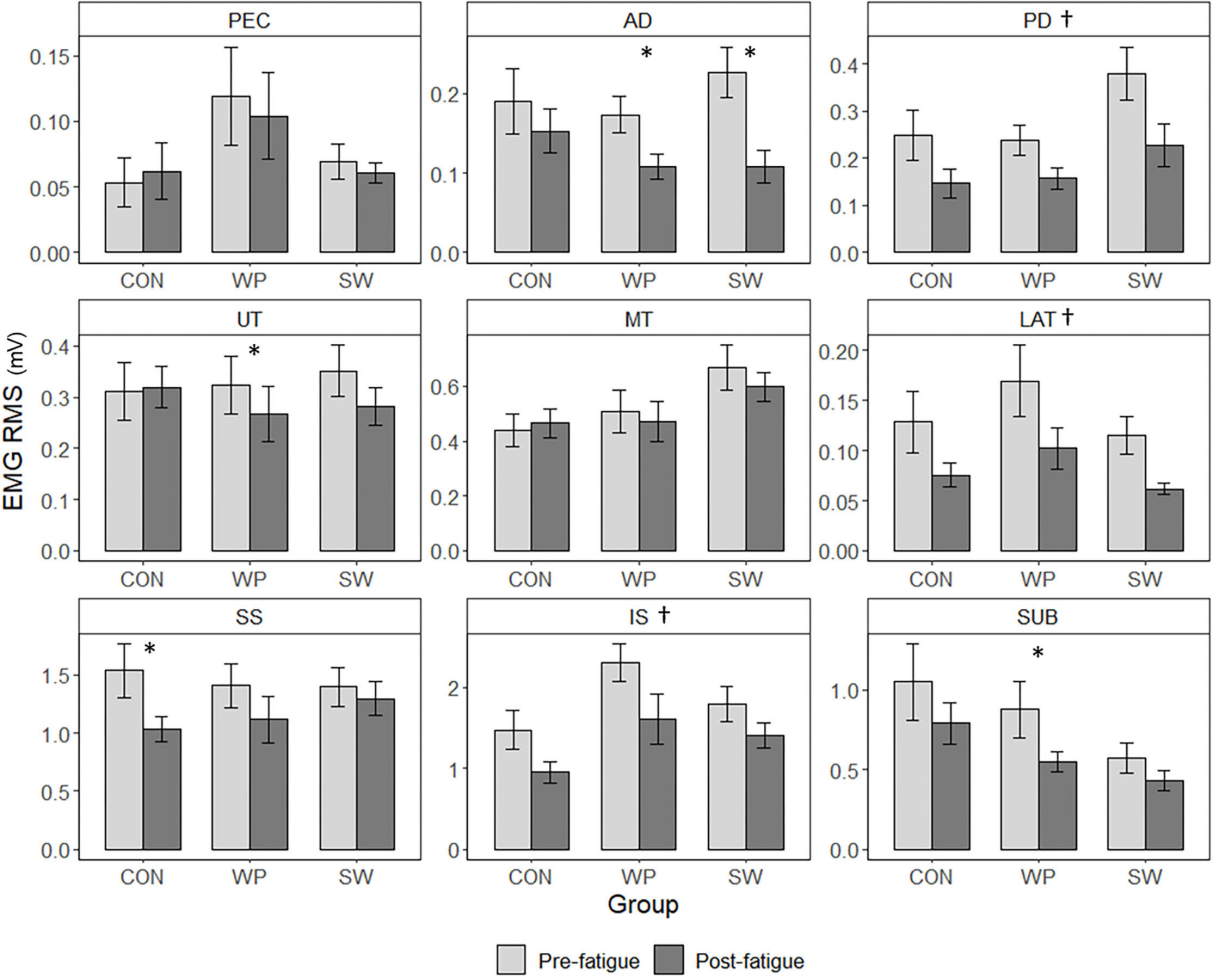
EMG RMS of water polo athletes (WP), swimmers (SW), and a control group (CON) were measured during maximal concentric external and internal shoulder rotations performed before and after a concentric shoulder internal rotation fatigue protocol. Values are estimated marginal means ± SE measured for pectoralis major (PEC), anterior deltoid (AD), posterior deltoid (PD), upper trapezius (UT), middle trapezius (MT), latissimus dorsi (LAT), supraspinatus (SS), infraspinatus (IS), and subscapularis (SUB). †Indicates a significant interaction of Muscle × Condition, with decreased RMS values after the fatigue protocol (*p* < 0.05). *Indicates a significant group-specific decrease in RMS (*p* < 0.05).

For EMG RMS measures during the MVCs, there were also significant Group × Condition × Muscle interactions $\left(X_{16}^{2}=80.033,\;p<0.0001\right)$ (Figure 5), indicating group-specific changes in activation. CON demonstrated a decrease in RMS for supraspinatus after the fatigue protocol (*p* = 0.005, *g* = 0.71); WP had lower post-fatigue RMS for anterior deltoid (*p* < 0.001, *g* = 0.91), upper trapezius (*p* = 0.046, *g* = 0.29), and subscapularis (*p* = 0.018, *g* = 0.67); and SW had lower post-fatigue RMS for anterior deltoid (*p* = 0.001, *g* = 1.37). Significant Condition × Muscle interactions $\left(X_{8}^{2}=111.863,\;p<0.0001\right)$ for EMG amplitude followed by pairwise comparisons indicated that, post-fatigue, all participants had lower RMS for posterior deltoid (*p* < 0.001, *g* = 0.731), latissimus dorsi (*p* < 0.001, *g* = 0.69), and infraspinatus (*p* < 0.001, *g* = 0.64). Hedges' *g* effect sizes for EMG RMS changes during the fatigue protocol and comparing the pre- and post-fatigue MVCs are listed in Table 3.

**Table 3:** Hedges' *g* effect sizes for changes in EMG RMS between the first minute (T1), middle minute (T2), and last minute (T3) of the internal rotation fatigue task, as well as between the pre- and post-fatigue maximal voluntary contractions (MVCs).

	**CON**				**WP**				**SW**			
	**T1 vs. T2**	**T1 vs. T3**	**T2 vs. T3**	**MVC**	**T1 vs. T2**	**T1 vs. T3**	**T2 vs. T3**	**MVC**	**T1 vs. T2**	**T1 vs. T3**	**T2 vs. T3**	**MVC**
Pectoralis major	−0.72*	−0.85*	0.01	−0.16	−0.60*	−0.41	0.12	0.13	−0.37	−0.28	0.00	0.54
Anterior deltoid	−0.37	−0.24	0.16	0.31	−0.10	−0.26	−0.17	0.91*	0.05	0.03	−0.03	1.37*
Posterior deltoid	0.33	0.91*	0.42	**0.66** ^†^	0.74*	0.90*	0.21	**0.81** ^†^	0.08	0.63	0.32	**0.80** ^†^
Upper trapezius	−0.03	−0.05	−0.01	−0.04	−0.07	0.01	0.06	0.29*	0.11	−0.01	−0.12	0.42
Middle trapezius	0.38	0.72*	0.35	−0.12	0.36	0.55*	0.20*	0.13	0.04	0.29	0.27	0.27
Latissimus dorsi	0.10	0.18	0.07	**0.56** ^†^	0.03	0.07	0.03	**0.69** ^†^	0.41	0.74*	0.44*	**0.97** ^†^
Supraspinatus	0.33	0.72	0.28	0.71*	0.26	0.85**	0.55*	0.42	0.22	0.43	0.18	0.17
Infraspinatus	0.30	0.34	0.04	**0.66** ^†^	−0.30	−0.02	0.30	**0.76** ^†^	−0.04	0.00	0.05	**0.54** ^†^
Subscapularis	−0.16	−0.14	0.01	0.33	−0.16*	−0.04	0.13	0.67*	0.11	0.11	0.00	0.49

*A negative value reflects an increase in EMG RMS.*

*Significant interaction of Muscle × Condition.*

*Significant group-specific change in RMS.

## Discussion

This study was the first to compare fatigue-related changes in shoulder muscle activation patterns between female athletes of different aquatic sports specialties. Among water polo athletes, swimmers, and controls, fatigue was induced by a repetitive shoulder internal rotation task performed at 50% MVC. WP had a shorter task duration than CON but also had higher pre-fatigue torque production than CON. SW and WP showed different patterns of muscle activation during the repetitive shoulder internal rotation task and as a result of the fatigue induced, and all groups demonstrated decreased shoulder stabilizer activation in the post-fatigue MVCs.

Both subjective and objective measures confirmed that fatigue was induced by the internal rotation task. All participants finished the task with higher ratings of perceived effort than when they started. Consistent with traditional definitions of muscle fatigue resulting from physical exertion (Gandevia, 2001), there was a reduction of force production capacity, as evidenced by lower maximal internal and external rotation torque values after the fatiguing protocol. Interestingly, CON performed the task longer than WP before reaching the termination criteria. While it may be initially counterintuitive that CON had greater endurance compared to high-performance athletes, this result may be attributable to strength differences between the groups, with WP producing significantly higher internal rotation torque than CON at baseline, even when accounting for weight. Water polo involves forceful and ballistic actions, and this type of training can promote shifts toward type II fiber types (Wilson et al., 2012; Plotkin et al., 2021), in addition to overall increases in muscle fiber cross-sectional area (Folland and Williams, 2007). On top of morphological adaptations to sports-specific training, neurological adaptations, such as enhanced motor unit firing rates, greater motoneuron excitability, and changes in inter-muscle coordination, may allow athletes to produce peak forces closer to their true maximum (Folland and Williams, 2007). In contrast, even when motivated, healthy but untrained individuals are less likely to be able to fully activate their agonists (Westing et al., 1988; Dudley et al., 1990). Thus, the target force of 50% MVC at which the fatigue protocol was performed may have been a lower intensity for CON compared to WP, relative to their true maximum. This would in turn result in a less physically demanding task for CON than it was for WP, allowing them to continue for longer. Although there have yet to be studies within female groups investigating if greater initial strength can be linked to higher fatigability, such associations have been made within the context of sex differences research. This suggests the presence of a strength-related mechanism to explain sex differences in fatigability and associated neuromuscular mechanisms (West et al., 1995; Hunter and Enoka, 2001; Hunter et al., 2006; Hunter, 2014).

EMG RMS changes during the fatiguing task were similar between CON and WP. Greater pectoralis major RMS for both groups, and greater subscapularis RMS for WP, at T2 compared to T1, suggest that there was an initial increase in agonist activation. There were no further increases at T3 for these muscles. Instead, there were decreases in RMS for posterior deltoid and middle trapezius for both groups, and supraspinatus among WP. Posterior deltoid, middle trapezius, and supraspinatus are all muscles that are more active in external rotation than in internal rotation (Boettcher et al., 2010; Gaudet et al., 2018a). Thus, as the fatiguing task continued, CON and WP may have used a strategy that first involved increased agonist activation, which was then followed by reduced antagonist activity. While this may have been a strategy to maintain task performance, concurrent activation of stabilizing muscles (co-contraction) is important for joint stability of the shoulder (Sangwan et al., 2014). Interestingly, there was comparatively little change in muscle activation among SW. Based on their sports expertise, they are also the group that would have been most habituated to such a repetitive cyclic task. Movement variability in locomotion is thought to be an adaptive mechanism that could reduce the risk of overuse injury (Bartlett et al., 2007). If this was a strategy more heavily relied upon by SW—who has expertize in repetitive, locomotor upper limb movements—fatigue-related changes in muscle activation among SW may not have been captured by comparing just the last five repetitions of the first, middle, and last minute.

There were also differences and similarities in EMG RMS changes for the MVCs. Decreases in RMS in maximal efforts may represent the fatigue of type II fibers (Cifrek et al., 2009) and/or an inability to sustain initially high firing rates (Gandevia, 2001). In the post-fatigue MVCs, although CON, WP, and SW all shared significantly lower EMG RMS for latissimus dorsi compared to the pre-fatigue condition, the largest effect size for this change was among SW. Interestingly, during the fatiguing task, SW was the only group to exhibit decreases in latissimus dorsi RMS—a muscle that, in swimming, is highly active in the pull-through (i.e., propulsive) phase of all four competitive swimming strokes (Nuber et al., 1986; Pink et al., 1991). In throwing, subscapularis is a major contributor to generating the forces needed for rapid acceleration, but also plays an important role in stabilizing the shoulder joint (Gowan et al., 1987; Escamilla and Andrews, 2009). Only WP demonstrated a significant decrease in subscapularis RMS and similarly, were the only group for which there were indications of subscapularis fatigue during the fatiguing task. Although more research is required to examine EMG parameters and their variability in parallel with fatigue development, these results hint that there is sports specificity underlying group differences in muscle activation between water polo players and swimmers during and after a fatiguing task.

It is also interesting to note that anterior deltoid RMS decreased among WP and SW, supraspinatus RMS significantly decreased among CON, and posterior deltoid and infraspinatus RMS were lower for all groups after the fatiguing task. Shoulder stabilizers have previously demonstrated neuromuscular fatigue induced by a repetitive, maximal, concentric internal and external rotation task (Gaudet et al., 2018a). The current findings suggest that a submaximal shoulder rotation task may also induce fatigue among the shoulder stabilizers. Infraspinatus and subscapularis activity is important for countering the superior shear force produced by supraspinatus and deltoid and minimizing the risk of subacromial impingement (Payne et al., 1997). Consequently, decreased anterior deltoid, posterior deltoid, and supraspinatus activation that occur in conjunction with decreases in infraspinatus and subscapularis RMS could potentially indicate a protective mechanism to maintain balance at the shoulder joint.

One limitation of this study is that the fatigue protocol may have been performed at different intensities relative to participants' true maximal strength. The length of time (~44 s) that the task was performed until the T1 data, and what occurred during the first 30 s (verbal feedback to help the participant maintain consistent torque output within the bandwidth) may have had a variable impact on T1 data, and as such, may also be considered as a limitation. Additionally, EMG has inherent limitations due to factors such as electrode placement, electrode movement, and individual anatomical differences. However, the same trained individual placed all electrodes in a secure fashion. As well, within-subject changes in EMG RMS were compared between groups, rather than one-time measures being compared between groups. Together, these steps reduce the impact of such factors on our measured outcomes. Group comparisons were also limited by the comparatively small sample sizes in each group. Finally, because of constraints related to athlete availability, the time since the last workout and the training phase of the season could not be controlled.

In conclusion, this was the first study to investigate fatigue-related changes in shoulder muscle activation among female groups with different aquatic sport specializations. When comparing the start, middle, and end of a repetitive internal rotation task, swimmers exhibited fewer changes in muscle activation compared to water polo players and controls, who appeared to use a strategy of initially increasing agonist activation and then reducing stabilizer activation to maintain performance. Patterns of EMG RMS in maximal shoulder rotations before and after fatigue was induced, as well as during the fatigue protocol, suggest that water polo players and swimmers may have had sports-specific ways of performing the same tasks. Whether one way was superior to another, or if they were simply different, remains to be determined with repeated testing and examining associations with injury development. As this is the first study to focus exclusively on female overhead athletes and their neuromuscular control, more research on these populations is needed to ultimately understand if there are sports-specific neuromuscular adaptations that are associated with injury development among female athletes, and in turn identify training and injury prevention strategies to promote healthy and continued sports participation among women.

## Data Availability Statement

The raw data supporting the conclusions of this article will be made available by the authors, without undue reservation.

## Ethics Statement

This study, which involved human participants, was reviewed and approved by the Centre de Recherche Interdisciplinaire en Réadaptation (CRIR-1247-0517). Written informed consent to participate in this study was provided by the participants' legal guardian/next of kin. Written informed consent was obtained from the individual(s) for the publication of any potentially identifiable images or data included in this article.

## Author Contributions

SK was the lead on research design, setup, recruitment, data collection, collaborated on analysis, and writing. LD led the data analysis and writing with the assistance of the other authors. MC assisted during research study design, recruitment, data collection, and analysis. JC actively supervised and advised on every step and decision made regarding the research study and completion of the project. All authors contributed to the article and approved the submitted version.

## Funding

This study was funded by the Natural Sciences and Engineering Research Council of Canada (NSERC RGPAS 477907).

## Conflict of Interest

The authors declare that the research was conducted in the absence of any commercial or financial relationships that could be construed as a potential conflict of interest.

## Publisher's Note

All claims expressed in this article are solely those of the authors and do not necessarily represent those of their affiliated organizations, or those of the publisher, the editors and the reviewers. Any product that may be evaluated in this article, or claim that may be made by its manufacturer, is not guaranteed or endorsed by the publisher.
